# Overview of the infant and young child feeding policy environment in Pakistan: Federal, Sindh and Punjab context

**DOI:** 10.1186/s12889-017-4341-5

**Published:** 2017-06-13

**Authors:** Hana Mahmood, Yasmeen Suleman, Tabish Hazir, Durre Samin Akram, Shahadat Uddin, Michael J Dibley, Saleem Abassi, Amara Shakeel, Narjis Kazmi, Anne Marie Thow

**Affiliations:** 1 Maternal, Neonatal and Child Health Research Network (MNCHRN)/ International Research Force (IRF), Islamabad, Pakistan; 2 Health, Education and Literacy Program (HELP), Karachi, Pakistan; 3 Maternal, Neonatal and Child Health Research Network(MNCHRN), Islamabad, Pakistan; 4 Children Hospital, Pakistan Institute of Medical Sciences (PIMS), Islamabad, Pakistan; 5 Complex Systems Research Group, The University of Sydney, Sydney, Australia; 6 School of Public Health, University of Sydney, Sydney, Australia; 7 Menzies Centre for Health Policy, School of Public Health, The University of Sydney, Sydney, Australia

**Keywords:** Malnutrition, Policy analysis, IYCF

## Abstract

**Background:**

Appropriate infant and young child feeding (IYCF) practices have been identified as important for appropriate child growth and development. (Ministry of Planning and Development, Ministry of National Health Services, Regulations and Coordination (2012)) Children in Pakistan still experience high rates of malnutrition, indicating a likely need for stronger IYCF policy. The purpose of this study was to identify major stakeholders who shape the IYCF policy environment and analyze which policies protect, promote and support IYCF practices, either directly or indirectly.

**Methods:**

This study was conducted at the federal level, and in the provinces of Sindh and Punjab. We identified policies relevant to IYCF using a matrix developed by the South Asian Infant Feeding Research Network (SAIFRN), designed to capture policies at a range of levels (strategic policy documents through to implementation guidelines) in sectors relevant to IYCF. We analyzed the content using predetermined themes focused on support for mothers, and used narrative synthesis to present our findings. For the stakeholder analysis, we conducted four Net-Map activities with 49 interviewees using the Net-Map methodology. We analyzed the quantitative data using Organizational Risk Analyzer ORA and used the qualitative data to elucidate further information regarding relationships between stakeholders.

**Results:**

We identified 19 policy documents for analysis. Eleven of these were nutrition and/or IYCF focused and eight were broader policies with IYCF as a component. The majority lacked detail relevant to implementation, particularly in terms of: ownership of the policies by a specific government body; sustainability of programs/strategies (most are donor funded), multi-sectoral collaboration; and effective advocacy and behavior change communication. Data collected through four Net-Map activities showed that after devolution of health ministry, provincial health departments were the key actors in the government whereas UNICEF and WHO were the key donors who were also highly influential and supportive of the objective.

**Conclusion:**

This analysis identified opportunities to strengthen IYCF policy in Pakistan through increased clarity on roles and responsibilities, improved multisectoral collaboration, and strong and consistent training guidelines and schedules for community health workers. The current policy environment presents opportunities, despite limitations. Our Net-Map analysis indicated several key government and international stakeholders, who differed across Federal and Provincial study sites. The detailed information regarding stakeholder influence can be used to strengthen advocacy.

## Background

High levels of malnutrition have serious consequences on the health and survival of infants and young children [1]. Almost 44% of children in Pakistan are stunted, 30% are underweight and 15% are severely wasted according to the National Nutrition Survey of 2011 [1]. One of the major reasons for such high rates of malnutrition is inappropriate Infant and Young Child Feeding (IYCF) practices which includes breastfeeding and complementary feeding [2, 3]. The prevalence of exclusive breastfeeding was 20.9% at 4 months and 12.9% at 6 months of age [1]. Similarly, although 51.3% mothers started giving semisolid foods to their children at the recommended 6–8 months of age, the proportion achieving minimum dietary diversity (children who received foods of 4 or more food groups) was only 3% [1].

Association of inappropriate feeding practices with malnutrition has been shown by various studies [4, 5]. The World Health Organization (WHO) and United Nations International Children’s Emergency Fund (UNICEF), with the broad participation of many stakeholders, developed the Global Strategy for IYCF to revitalize global commitment for improving infant and young child nutrition by promoting, protecting and supporting appropriate IYCF [6]. Effective interventions for IYCF are well established, including but not limited to interventions for skilled support by the health system, interventions for community based counseling /support and IYCF in emergency circumstances. Additionally, training and capacity building of community health workers to deliver IYCF support and intervention is also imperative [7–10]. But for the best outcomes at national level, such interventions need to be supported by appropriate policies. And the development of these policies is influenced by the major stakeholders of the policy environment including the ministry, nutrition officers, academicians, researchers, development partners, etc. It is important to understand how different actors come together and influence policies and their implementation in the real world [10].

The health policy environment of Pakistan has been markedly influenced by its unsteady political environment. The National Ministry of Health was devolved in 2011 in the wake of the 18th amendment of the constitution [11]. As a result, a number of federal health responsibilities were placed under the jurisdiction of other government ministries/divisions. The provinces were empowered to operate their health systems. One of the major outcomes was that health policy formulation and planning was also devolved to the provinces along with service delivery leading to likely differences in health policies between provinces [11].

The aims of this study are to 1) identify and analyze policies in Pakistan that protect, promote and support IYCF practices, and 2) identify key stakeholders who shape the IYCF policy environment, through technical and/or financial contributions. We conducted this analysis at the Federal level and in Sindh and Punjab. Sindh and Punjab are the most populous provinces and could be considered as representatives of the policy making process in the country. With respect to the health policy environment of Pakistan, this analysis will enable us to understand shortcomings in the existing policies focusing on IYCF (directly or indirectly), in order to identify opportunities which could strengthen future policy formulation, implementation, and advocacy [12].

This study is a part of a research project on IYCF policies by South Asian Infant Feeding Research Network (SAIFRN) involved in mapping and analyzing policy initiatives for infant and child nutrition in Bangladesh, Pakistan, India, Nepal, and Sri Lanka in collaboration with University of Sydney, Australia.

## Methods

A descriptive research design was employed to conduct this study whereby policy content analysis was done along with net map activities to collect quantitative and qualitative data. The purpose of coupling content analysis with net mapping was to assess what the policies mentioned regarding the research question and identify its association with what is actually happening on the ground. The qualitative research design was based on grounded theory.

### Data collection

#### IYCF policy content analysis

Policy has been defined as ‘the process by which governments, institutions or organizations translate their political vision into programs and actions to deliver ‘outcomes’ – desired changes in the real world’ and ‘decisions taken by those with responsibility for a given area… broad statements of goals, objectives and means that create the framework for activity’ [12, 13].

Policies, strategies, guidelines and relevant documents were included in this analysis. “Appropriate IYCF practice” has been defined as initiation of breastfeeding **within 1 h of birth**, exclusive breastfeeding for the first six months of life and introduction and continuation of safe and nutritionally adequate foods while breastfeeding is continued for up to 2 years of age [6].

The SAIFRN core policy team used a mind mapping approach to identify relevant policy document types and sectors that possibly have support for IYCF ranging from whole-of-government strategic policy documents to implementation guidelines relevant to IYCF. The team also developed a matrix to guide data collection, with four domains for assessing policy content based on best-practice recommendations for interventions to support appropriate IYCF. These criteria were also used as themes for coding:

1. General Support for IYCF;
2. Provision of standardized information to mothers and caretakers;
3. Training of health workers to counsel for IYCF;
4. Enabling mothers / caregivers to engage with best practice interventions

Relevant policy documents were identified through searching government websites and meetings with relevant government stakeholders in Sindh and Islamabad (Box 1). Although as many documents as possible were obtained, pertaining to the subject, however, there are chances that a few would have been missed due to inaccessiblity.

### Data sources

The following were the main sources of data for this study:

- Ministry of National Health Services, Regulations and Coordination.
- Nutrition Cell, Department of Health, Sindh.
- Website of the government of Punjab
- Website of the government of Sindh
- Website of the planning commission of Pakistan
- Website of the finance ministry of Pakistan
- Website of the Ministry of Social Welfare.
- Website of the planning and development department, Punjab
- Planning and Development Department, Sindh
- Website of Technical Resource Facility
- Website of WHO
- Website of the Pakistan humanitarian Response
- website of the Punjab Health Sector Reform Unit [14–32]

For each policy document, the following data was entered into the matrix:

- Any mention of nutrition and specifically IYCF
- Name of the Policy
- Year of release
- Whether it is endorsed by Cabinet or not
- Mentions of other relevant policy documents (note under the policy that is supported/referred to – e.g. Note under the National Nutrition Policy if it is supported by the National Development Strategy)

The review period was from May 2013 to May 2014. Two reviewers performed screening of documents obtained.

### Stakeholder analysis

#### Data collection tool

Stakeholder analysis was conducted using the Net-Map method, developed by the International Food Policy Research Institute (IFPRI) [33]. Net mapping is an interview based mapping technique to understand, discuss, and visualize how various actors influence focused outcomes through various links [33]. The SAIFRN Policy study team developed an interview guide during training, defining the terminologies used in the research question as follows:

- ▶ “Protect” was defined as policies creating an environment which protects mothers from misinformation and from competing demands during the early period of their baby’s life (for example, maternity leave legislation).
- ▶ “Promote” was defined as people have an understanding of the importance of appropriate feeding in the first 24-months of life.
- ▶ “Support” meant policies that create an enabling environment for mothers to choose appropriate infant feeding practices.
- ▶ Financial Link/support was defined as exchange of funds or in-kind support related to IYCF in the form of funding or supplies for programs or staff employed by the recipient, or budget provision.
- ▶ Technical Link/support was defined as formal exchange of technical support, training, technical guides, equipment, staff/consultant (preselected), information, research evidence, routine data, field experience/case studies.

The interview asked about: actors that influence policies and programs for IYCF; the links between actors (both technical and funding); level of influence; and level of support for IYCF. Participants were engaged with an interactive group discussion and physical mapping of the data obtained through consensus. Each activity was audio recorded upon taking consent except for one where respondents refused audio recording. It was conducted by a moderator, an observer and two note takers and lasted for 4–5 h. The recorded activities were transcribed and translated into English.

The interview guide and method was pre- tested with experts from Sindh, Punjab and Federal capital with knowledge on IYCF policy environment who were not included in the sample and the interview guide refined for local context.

### Respondent categories

Potential categories of respondents were developed by the Pakistan SAIFRN team, based upon knowledge of stakeholder involvement in IYCF policy environment. The key stakeholder categories identified were those belonging to the Government, Development partners, Non-government organizations, academic and research institutions, and others. Purposive sampling was conducted to recruit respondents who were knowledgeable about IYCF policy environment in Pakistan and key stakeholders were identified. Initially they were contacted by phone and thereafter letters and emails were sent to them to request participation.

### Number of net-map activities

A total of four Net-Map activities were conducted, with 49 participants One of the activities was conducted to identify stakeholders in the federal context, two in the context of Punjab and one of Sindh. The two Net-Map activities held in Punjab were to facilitate attendance of maximum number of participants as they were conducted in two separate locations Table 1.

**Table 1:** Net-Map Activity Stakeholder [40]

Stakeholders	(Federal)	(Punjab)	(Sindh)
Government Sector - Health	3	4	1
Government sector –Non Health	1	2	2
Research and Academic	2	5	
Development partner	3	8	4
NGO /Civil Society	1	7	2
Other		2	2
Total	10	28	11

### Analysis

#### IYCF policy content analysis

Within each group of policies based on the domains, we adapted narrative synthesis and descriptive analysis approaches for policy content analysis. Narrative/thematic synthesis involves describing the findings of an analysis or review using an integrated critical perspective [34]. The steps taken to conduct the synthesis included development of the theoretical model revolving around the IYCF policy environment, preliminary synthesis using the matrix, exploring associations in the data, and assessing robustness of the synthesis results. We used this to describe the content related to IYCF, within the umbrella of nutrition more broadly, for each policy document identified. This narrative synthesis approach enabled us to consider the specific policy content within the broader policy context, in relation to supporting IYCF best practice, and to engage with the complexity of the policy landscape.

### Stakeholder analysis

#### Software

Net maps generated as a result of the activities were incorporated into an excel sheet and prepared for analysis by ORA (Organizational Risk Analyzer, copyright Carley, Carnegie Mellon University), a specialized software meant for analysis of Net-Map data. Four network centrality measures were quantified to identify linkage between different actors and the support provided therein (technical and financial) as follows:

In-degree: Degree centrality quantifies the number of connections an actor has. This measure quantifies the tendency of an actor to receive ‘choices’ from the other network actors [33]. Here, ‘choices’ indicate the intention of other actors to form link with the actor under consideration. In other words, in-degree is a measure of receptivity or popularity. The arrow coming in a diagram indicates this measure.

Out-degree: This measure quantifies the tendency of an actor to make ‘choices’ in terms of forming links with other network actors [33]. In other words, out-degree is a measure of expansiveness or activity of an actor in a network. The arrow going out indicates this measure.

Betweenness: This centrality measure represents the capacity of an actor to control the flow of information between any pair of all other member actors in a network [33].

Closeness: Closeness centrality represents the reachability of an actor from the other actors in a network [33]. An actor is said reachable from another actor if there is a path linking the two actors.

### Qualitative data analysis

Using Axial coding technique, transcripts and notes from the respective Net-Map activities were coded for themes related to the IYCF policy environment, with a focus on: which actors were included as stakeholders and why; avenues and reasons for influence of actors; and how stakeholders provided technical and financial support for IYCF policy. In axial coding, instead of focusing on the text and extracting codes, the analysts used their predefined themes to develop a code book based on the research question. This coding was done manually and subsequently a code book was developed by two researchers (HM and YS) who were trained on qualitative data analysis. These qualitative data were used to interpret and explain the quantitative net map findings.

## Results and Discussion

### Policy content analysis

#### Overview of policies

We identified 38 documents based on the study objective from the sources listed above. These were documents that focused on nutrition as a whole in local and global context where Pakistan was mentioned in the policies/strategies/programs/papers. Out of these, 19 met our inclusion criteria of covering some or all of the key domains identified. Eleven of these were nutrition and/or IYCF focused and 8 were general broader policies with IYCF as a component. Ten policies were excluded due to being the older versions of the new published policies, and the rest were not relevant to identified key domains for IYCF.

**Fig. 1:**
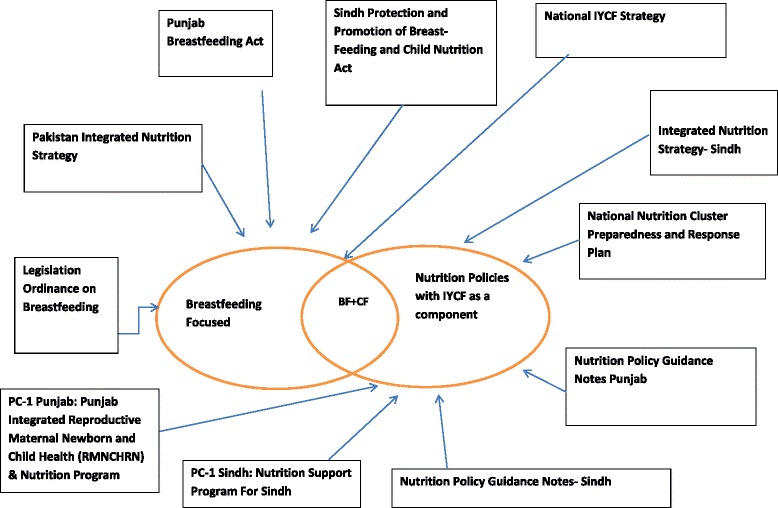
IYCF Related Policies

Of the 11 nutrition policy documents identified, five focused exclusively on IYCF (four exclusively on breastfeeding) and six mentioned IYCF but were broadly nutrition focused (Fig. 1). Although appropriate complementary feeding is essential for good nutrition, only one IYCF-specific document included complementary feeding.

**Fig. 2:**
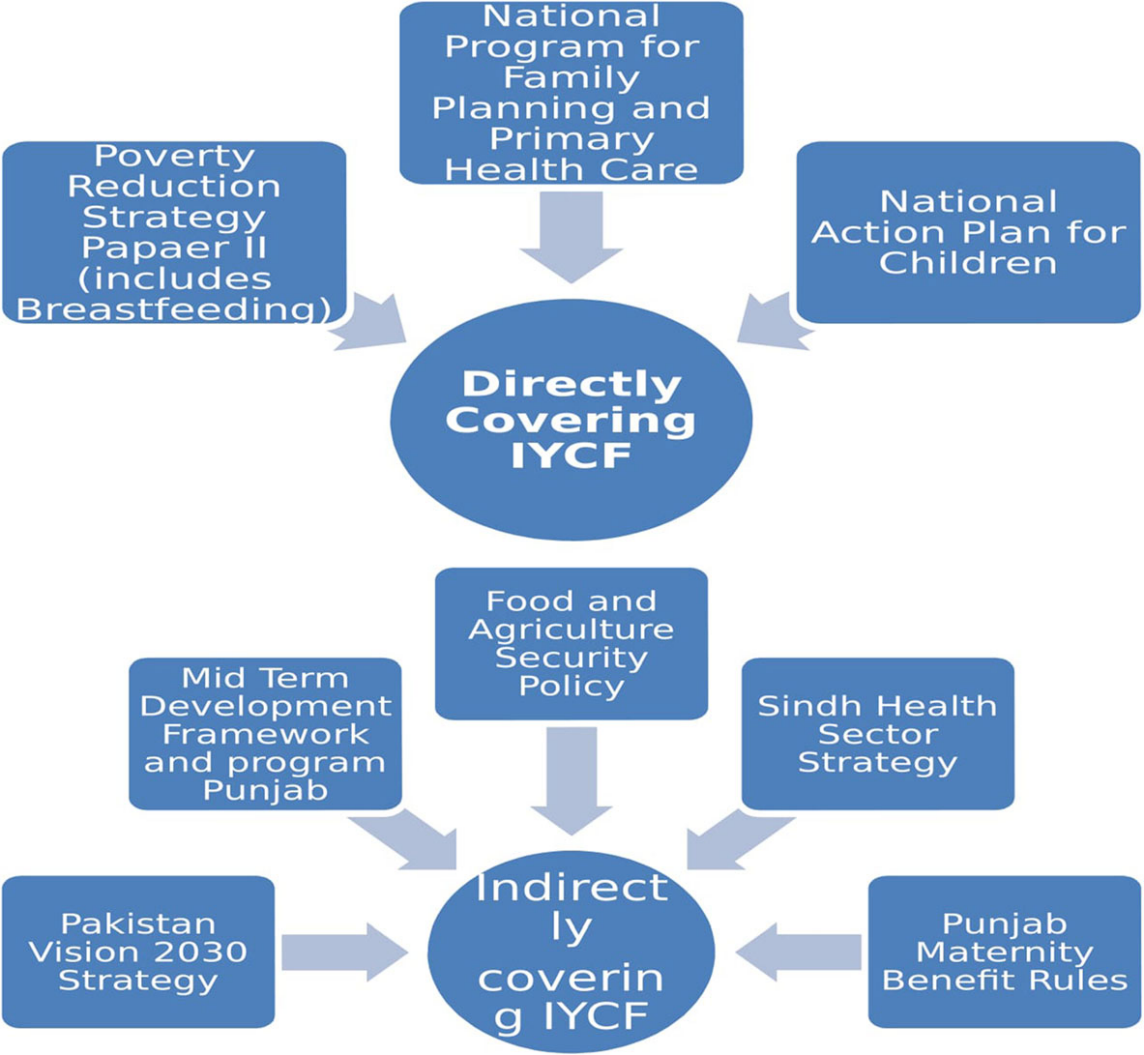
Generalized Policies/Strategies/Program directly and indirectly covering IYCF

Eight policies covered broader multi-sectoral issues and had nutrition and/or IYCF as a component. Three policies directly addressed IYCF, and 5 indirectly focus on IYCF by mentioning another program or broadly covering child nutrition with not much detail on IYCF (Fig. 2).

In the following sections, we present results of our policy content analysis in line with the four domains detailed above.

### General or strategic support for IYCF

General or strategic support for IYCF is present in the form of: establishment of advisory committees; integration of IYCF into health service delivery; and support for food security and access (Table 2).

**Table 2:** IYCF Policy Documents with Respect to Four Domains

Policy	Description of Policy -	Support for IYCF					Further details on IYCF in policy
		General support for IYCF)	Provision of correct information to mothers	Training of health workers to counsel mothers	Enable mothers to engage with health care workers	Other aspects related to IYCF but not falling in these four domains?	
NATIONAL							
The Protection of Breastfeeding and Child Nutrition Ordinance, 2002 [14]	The ordinance is based on international code of marketing of breast milk substitutes.	✓	✓			Penalties for promotion of breast milk substitutes	After devolution it has been adopted by individual legislations at the Provincial levels with Punjab and Sindh amongst the first of the provinces to adopt it.
Pakistan Vision 2030 strategy (Planning Commission, 2007, approved by the National Economic Council) [24]	Encompasses inter sectoral efforts to improve malnutrition like health and nutrition, education and social development	✓					In article 6 there is a section on improving food security. Increase in food availability of the food will increase the diversity of the complementary food
Poverty Reduction Strategy Paper II (2008–2010, Finance Division, Government of Pakistan) [21]	Includes development of a practical program to reduce child under-nutrition.	✓					Includes promotion of breast feeding and development of a programme for improving nutrition status of children under 3 years
Food and Agriculture Security Policy (2013, Ministry of National Food Security and Research) [25]	The key elements of the policy is access of food to children and women through measures like strengthening education of nutrition	✓					Increased availability of food at the household level will increase the diversity of the complementary food.
Pakistan’s Integrated Nutrition Strategy (PINS, 2009–2010) [30]	The goal is to have a well-nourished nation with sound human resource that effectively contributes to the National Development and Prosperity.	✓	✓	✓			Increase the knowledge and skills of service providers, caregivers, households and communities in IYCF through communication strategy and civic education. Provide knowledge and skill to service providers, in nutrition management and IYCF through in-service and on job trainings. Up-scaling micro-nutrients (sprinkles) to children in line with CMAM interventions
National Strategy for Infant and Young Child Feeding in Pakistan (Ministry of Health- 2008) [18]	Institute of Public Health and Nutrition and Ministry of Health and Family Welfare are the responsible bodies. Implementation level policy support	✓	✓	✓			To improve the nutritional status, growth and development, health and survival of infants and young children through optimal IYCF practices
The National Nutrition Cluster Preparedness and Response Plan (2014) [27]	It is a common framework to guide the actions of all partners in the nutrition sector in the event of a disaster. It is a flexible and dynamic document that will be updated based on lessons learnt in future emergency responses.	✓		✓			Protection and promotion of appropriate IYCF practices through strengthening caring capacity of family members, and health care provider at community and facility levels; and protection of breastfeeding
National Action Plan for Children (2007) [23]		✓		✓			Promotion of Breast Feeding, exclusive breast feeding, supplementary feeding and caring practices at community level through media, HCP including LHWs Growth monitoring and maintaining growth charts.
National Program for Family Planning and primary healthcare; The Lady Health Workers Program(2010–2015, Ministry of Health) [29]	Approved by the Cabinet the programme runs through a network of community health workers	✓	✓	✓			Counselling of mothers on IYCF by lady health workers.
PUNJAB							
PC-1 Punjab; Punjab Integrated Reproductive Maternal Newborn and Child Health (RMNCHRN) & Nutrition Program 2013–2016. [18]	To be implemented by Health Department with departments of Food, Agriculture, Livestock and Education.	✓	✓	✓	✓		Increasing the proportion of children 6–23 months fed in accordance with all 3 IYCF practices (food diversity, feeding frequency, consumption of breast milk or milk), up to 40% in 2016.
Mid Term Development Framework and Program Punjab (2014–17, Government of Punjab) [20]	Focus on reduction of nutrition generally in Punjab through an integrated maternal, newborn and child health program						Includes amongst various interventions an Integrated Reproductive, maternal, newborn and child health and nutrition program.
Nutrition Policy Guidance Notes- Punjab (2012, Government of Punjab) [26]	Guidelines for protocols to be followed for IYCF and CMAM	✓	✓		✓		Increase measures to promote exclusive breastfeeding for 6 months, dietary diversity for young children, intensified self-care and IYCF counselling of pregnant women and mothers at the community and facility levels.
Punjab Protection of Breast-Feeding and Child Nutrition (Amendment) Act 2012. [15]	Legislation approved by the Provincial Assembly Punjab. Based on international code of marketing of breast milk substitutes. High level support. It has replaced ‘The Protection of Breast- Feeding and Child Nutrition Ordinance, 2002’	✓	✓				Dissemination of information and educational materials on infant feeding.
Punjab Maternity Benefit Rules [28]	Approved by the Provincial Assembly of Punjab. It has replaced “Punjab Maternity Benefit Ordinance,1958”	✓				Provision of leave to breastfeed children	Although the act does not mention IYCF practices but indirectly it does show support to IYCF in a sense that it enforces organizations to provide a 6 weeks pre and postnatal paid leave to any woman who has worked in that organization for more than 4 months. However there is no mention of availability of feeding rooms.
SINDH							
PC-1 Sindh; Nutrition Support Program for Sindh (NSP) (2013)	The overall goal of the Nutrition Support Programme Sindh (NSP) is to improve the nutritional status of children less than 5 years and that of pregnant and lactating women, with a priority focus on malnourished.	✓	✓✓	✓	✓		Includes training of community health workers on IYCF, and lactation management.
Nutrition Policy Guidance Notes- Sindh.		✓	✓		✓		Intensified counselling of pregnant women and mothers on IYCF at the community and facility levels reinforced by media and mobile phones.
Health Sector Strategy Sindh 2012–2020 [20]	Guides the operational plans of medium and long term programs and projects with a special focus on nutrition. Mainstreaming key evidence based nutrition interventions through health sector and coordination with other department on nutrition as part of a larger provincial inter-sectoral strategy on nutrition inclusive of micro-nutrient supplementation, community based awareness and counselling	✓	✓✓				Counselling of mothers by HCP
The Sindh Protection And Promotion Of Breast- Feeding and Child Nutrition Act, 2013. [16]	Legislation approved by the Provincial Assembly Sindh. Based on international code of marketing of breast milk substitutes. It has replaced ‘The Protection of Breast- Feeding and Child Nutrition Ordinance, 2002’	✓	✓				Dissemination of informational and educational materials on infant feeding.
Integrated Nutrition Strategy Sindh	The strategy aims to promote health and quality of life through an inter-sectoral approach to food and nutrition, environment, education, social protection and health in Sindh province so that it contributes to the reduction of stunting in children aged 0–24 months by 10 percentage points (from an estimated 49.8% to 39.8% by the end of 2016.	✓	✓✓	✓			Target audience includes minus 9 months to 2 years children. Training of HCP in communities on IYCF

The Breastfeeding Ordinance, the Punjab and Sindh Provincial Breastfeeding Acts and the National IYCF strategy all provide for the development of the national and provincial nutrition boards and committees with a mandate to advise the Federal Government and provinces on development of policies for the promotion and protection of IYCF [14–17]. More than half of the members of these boards are required to be qualified professionals in infant and young child nutrition with at least one member from the industry involved in manufacturing of designated products. This shows that the emphasis of the individuals involved is limited to only those qualified for IYCF with no involvement of other sector stakeholders like agriculture, social welfare etc. The roles and responsibilities of these committees include receiving reports of violations of the acts/rules, recommendation of investigation of cases against manufacturers, distributors or health workers violating the acts/rules, to plan and disseminate IYCF related material to health workers, and to propose guidelines on any relevant IYCF matter as indicated in the acts. These details however, are generalized and are not specific to the provincial context which is important as the devolution has provided autonomous powers to each province.

The document on Planning Commission-1 Punjab, Mid Term Development Goals and Poverty Reduction Strategy Paper II ensure that promotion of breastfeeding is integrated into health care service delivery, starting from the health facility where a child is born, to the household where community health workers visit the mothers on monthly basis [18–22].

General policy support for appropriate IYCF is also present in the National Action Plan for Children, Pakistan Vision Strategy 2030, Food and Agriculture Security Policy, and Nutrition Policy Guidance Notes, which support increased availability of food at the household level and increasing the diversity of complementary food through interventions that are directed towards agriculture and related development policies [23–26].

### Provision of correct information to mothers

Policy support for the provision of correct information to mothers included consistent terminology and information regarding best IYCF practices, and avenues for providing information to mothers through various sources like Lady Health Workers/Community Health Workers and media.

Key concepts and terms, including early initiation of breastfeeding, exclusive breastfeeding till 6 months of age, appropriate and timely complementary feeding, are defined clearly and consistently in the National Ordinance on Breastfeeding and the National IYCF Strategy [14, 17]. The National Ordinance also recommends that a National Infant Feeding Board is constituted, which has responsibility for dissemination of correct information through health workers. However, how, when and through whom to disseminate this information, has not been laid out. These initiatives also support the specific IYCF communication strategies outlined in National Action Plan for Children and Nutrition Policy Guidance notes, which use, for example, mobile phones for disseminating information [23, 26]. But the workflow process to achieve the targets set in these strategies has not been outlined. There is also specific policy support for efforts to communicate with the poorest households and induce behavior change. This policy support is designed in a way to effectively communicate with illiterate and less educated mothers, as highlighted in the Nutrition Policy Guidance Notes, but it does not inform what means of communication is to be used along with the content and frequency to communicate. Additionally, there were financial mechanisms explained in these documents to achieve the said objectives. Each province has a separate social and cultural context, however the means and content of communications have not been highlighted in any of the documents. This is required in the post devolution period since each province is responsible to modify the national policies per their requirement.

We also identified specific policy support for enabling mothers to follow proper IYCF practices in case of emergencies as indicated in the National Cluster Preparedness Response [27]. Additionally, the Punjab Maternity Benefit Rules also emphasized on the provision of correct information to mothers but it did not highlight the content of that information and did not include the informal sector women workers [28].

#### Training of health workers to counsel women

Although there are 10 documents in this group that cover the training component for IYCF, however, only one of these policies (National Program for Family Planning and Primary Health care) includes detailed information on the training schedule, training content, number of workers to be trained, trainers and their qualifications, budget allocated on trainings, monitoring of trainings, and venues of training [29]. The rest of the nine documents only highlight the importance of capacity building and trainings of the lady/ community health workers by increasing the knowledge and skills but have incomplete detail on:

✓ how, when, and where the trainings are to be provided

✓ the content of the information to be provided to the mothers and

✓ The trainer profile

✓ Updating the training curriculum

✓ Defining the roles and responsibilities of the training implementation agencies

✓ Monitoring and evaluations of those trainings

These documents include the Pakistan Integrated Nutrition Strategy, National Cluster Preparedness Response, Breastfeeding Ordinance, National IYCF Strategy, Sindh and Punjab Breastfeeding Acts, Sindh and Punjab PC-1 s, Sindh Integrated Nutrition Strategy [15, 16, 18, 27, 30, 31].

#### Enabling mothers to engage with care

Policies that enable mothers to engage with health care workers included provisions for maternity leave and community-based access to health services supporting IYCF.

Upon the health ministry devolution the West Pakistan Maternity Benefit Ordinance 1958 was repealed as a Federal law and was to be re-enacted as a provincial law [32]. Yet to date, of all the provinces, only Punjab has incorporated this law in its provincial government, developing the Punjab Maternity Benefit Rules as a replacement to that ordinance. These rules provide 6 weeks pre and postnatal paid leave to any woman who has worked in any organization for more than 4 months. These rules apply to the whole of Punjab and include all categories of organizations whether government, non-government, industrial or commercial excluding tribal areas. In addition to the maternity benefit, the rules indicate that the woman shall also receive a separate medical bonus. It also mentions two nursing breaks of 20 min duration apart from other usual breaks in a feeding room if available or additional 15 min if the woman needs to feed at home. It did not however mention benefits for informal workers of the province and only focuses indirectly on breastfeeding with no importance highlighted about the benefits of proper complementary feeding [28].

The National Action Plan for Children and the National Program for Family Planning and Primary Health Care indicated facilitation of health care access through lady health workers where mothers were unable to go the facilities due to any reason. This service extended to those women living in rural communities. However, there were no details on the kind of special provisions for these women or how the facilitation was to be achieved [23, 29].

### Stakeholder analysis

A large number of actors were identified during the Net-Map activities; however, there were only a few actors which were strongly influential over the IYCF policy environment. As mentioned earlier, the data collection and analysis has been done while keeping the overall IYCF scenario in mind, irrespective of the devolution of the health ministry. Both retrospective and prospective links and actors have been identified. The following analysis provides information for the Federal, Punjab and Sindh level separately for both the technical and funding links.

In interpreting the figures, each circle on the map reflects the actor named by the participants and lines between actors depict the links either technical or financial. The size of the circle for each actor on the map depicts their relative influence in relation to the objective. The larger the size of the circle is, the more influential that actor is. The integer value against each actor reflects the level of support for that actor. A value of 2 means highly supportive, 1 means supportive, 0 means neutral and −1 means competing interests. Here competing interest is anything or any body that interferes with, or could reasonably be perceived as interfering with, the objective of supporting and/or promoting IYCF. The arrow direction indicates the support provide to and from an actor. All links between stakeholders are valued.

### Federal

**Fig. 3:**
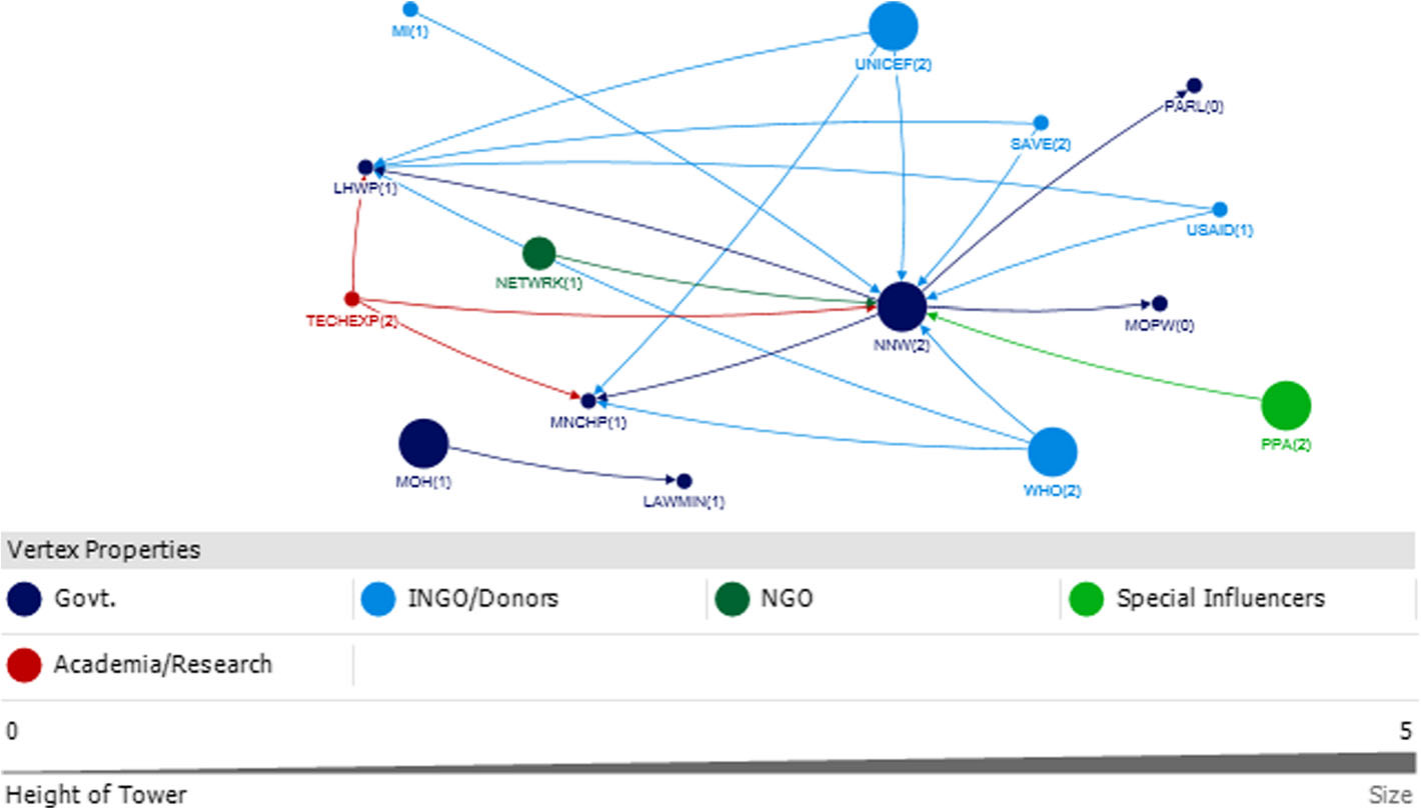
Federal Technical Network

**Fig. 4:**
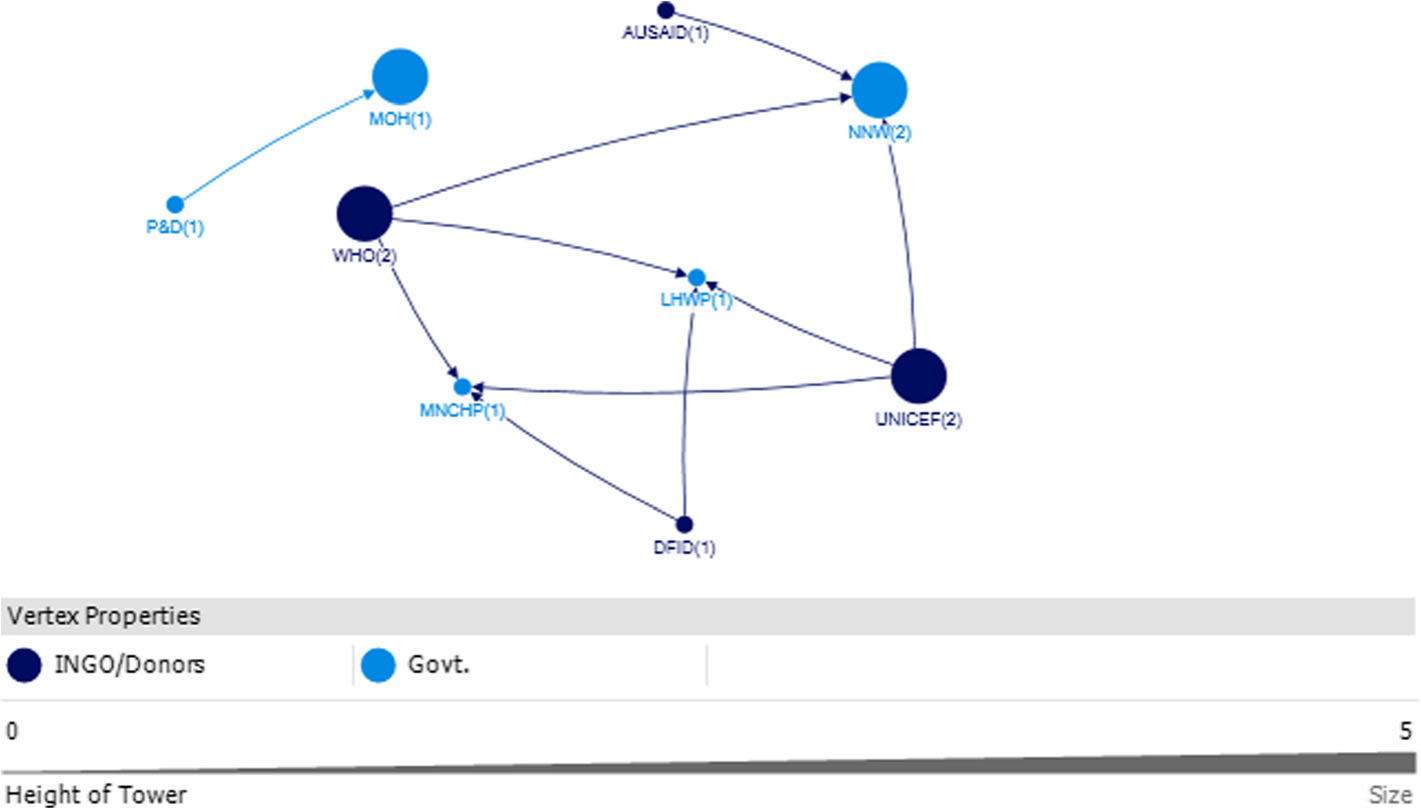
Federal Funding Link

A total of 15 actors were identified in the technical network and 9 were identified in the funding network in the federal area (Figs. 3 and 4).

### Technical federal

With respect to technical support, the National Nutrition Wing (NNW) was identified as the core and highly influential actor, central in the IYCF policy landscape in the federal capital. The NNW received a high level of technical support (in-degree), provided technical support (out-degree), was accessible to other actors in the network (closeness centrality), and was influential over the flow of information between actors by its position in the network (betweenness centrality). (Table 3, Fig. 3). UNICEF and WHO were the most highly influential development partners, through provision of technical support (out-degree), accessibility to network actors (closeness centrality) and control over information flows (betweenness). In all the figures the links between the Ministry of Health and Planning Commission represent the period before the devolution of the health ministry. Although the ministry of health has been devolved but the Planning Commission still exists however, now it has links with the respective provincial health departments.

**Table 3:** Top 10 actors in technical and funding support network at federal level with respect to four network measures

Federal								
Rank	Technical				Funding			
	In-degree	Out-degree	Between-ness	Closeness	In-degree	Out-degree	Between-ness	Closeness
1	8 NNW	4 NNW	103.500 NNW	0.083 NNW	3 LHWP	3 UNICEF	10.667 NNW	0.111 UNICEF
2	6 LHWP	3 TECHEXP	9.000 LHWP	0.060 MOH	3 MNCHP	3 WHO	6.667 UNICEF	0.111 WHO
3	4 MNCHP	3 UNICEF	2.000 MNCHP	0.060 LAWMIN	3 NNW	2 DFID	6.667 WHO	0.11 P&D
4	1 LAWMIN	3 WHO	0.500 TECHEXP	0.056 LHWP	1 MOH	1 AUSAID	4.667 LHWP	0.11 MOH
5	1 MOPW	2 SAVE	0.500 UNICEF	0.050 MNCHP		1 P&D	4.667 MNCHP	0.1 NNW
6	1 PARL	2 USAID	0.500 WHO	0.048 TECHEXP			0.667 DFID	0.1 LHWP
7		1 MI		0.048 UNICEF				0.1 MNCHP
8		1 MOH		0.048 WHO				0.077 DFID
9		1 NETWRK		0.045 SAVE			0	0.067 AUSAID
10		1 PPA		0.045 USAID				

Refer to list of abbreviations

The other actors influential through provision of technical support were technical experts, the Ministry of Health, Pakistan pediatric association and non profit bodies like the Micronutrient Initiative, The Network and Save the Children (Table 3). All actors identified as providers of technical support were seen to be supportive of IYCF objectives Fig. 3.

### Funding federal

The main providers of funding (out-degree) at the Federal level were international agencies and donors (Fig. 4; Table 3). UNICEF and WHO were most influential, also in terms of their strategic position in the network (closeness and betweenness). A few key government actors were influential through their role in allocating and initiating funding. The (qualitative) discussion indicated that although the Planning Commission was involved in funding, it was the Ministry of Finance that was responsible for the allocation of funds for IYCF.

The National Nutrition Wing, the Lady Health worker program and the MNCH program were the main receivers of funding (in-degree), and also had influential roles as a result of their accessibility (closeness) and degree of control over the network (betweenness).

### Actor communities (federal)

**Fig. 5:**
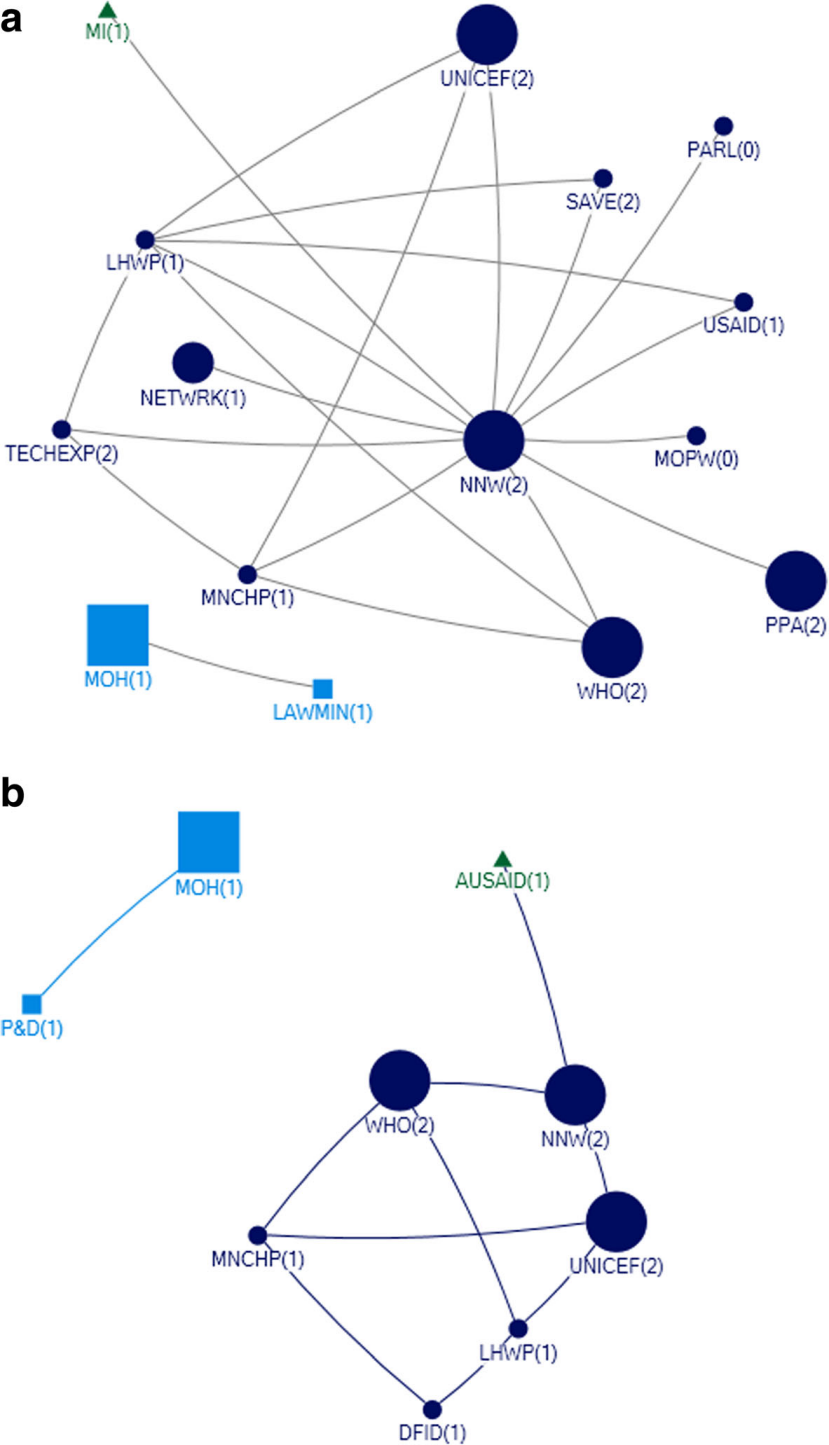
Federal Technical and Funding communities

A community indicates that actors (belonging to that community) have more connections (or collaborations) among themselves compared to their connections with the other actors. At the Federal level, there was one major actor community for funding, and another for funding (Fig. 5). Each of these was diverse, encompassing government and non-government actors. The qualitative discussion indicated that the major communities reflected the most influential actors in the post devolution era, indicating that after devolution there was more cohesiveness among influential actors in technical support network and funding network, respectively, at federal level.

### Punjab

**Fig. 6:**
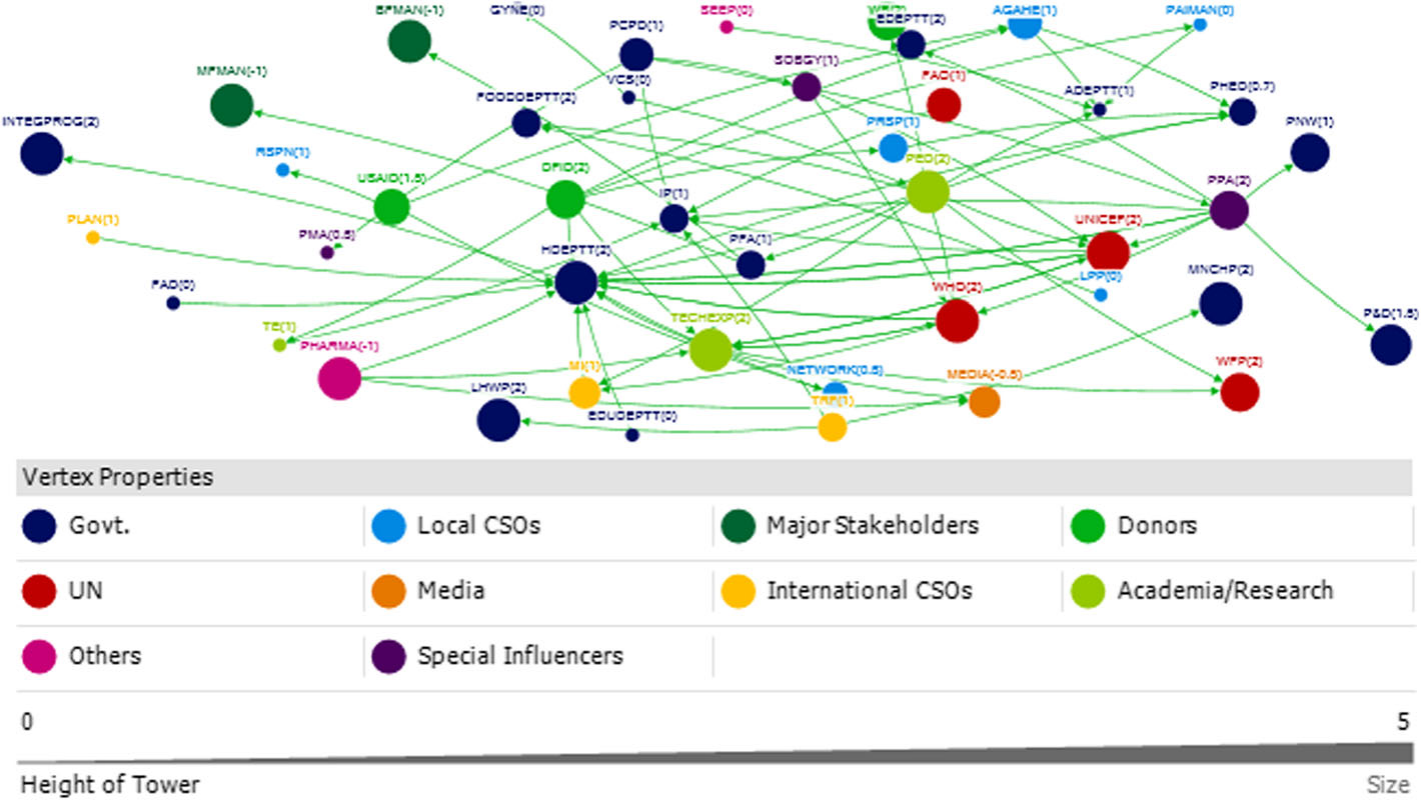
Punjab Technical Network

**Fig. 7:**
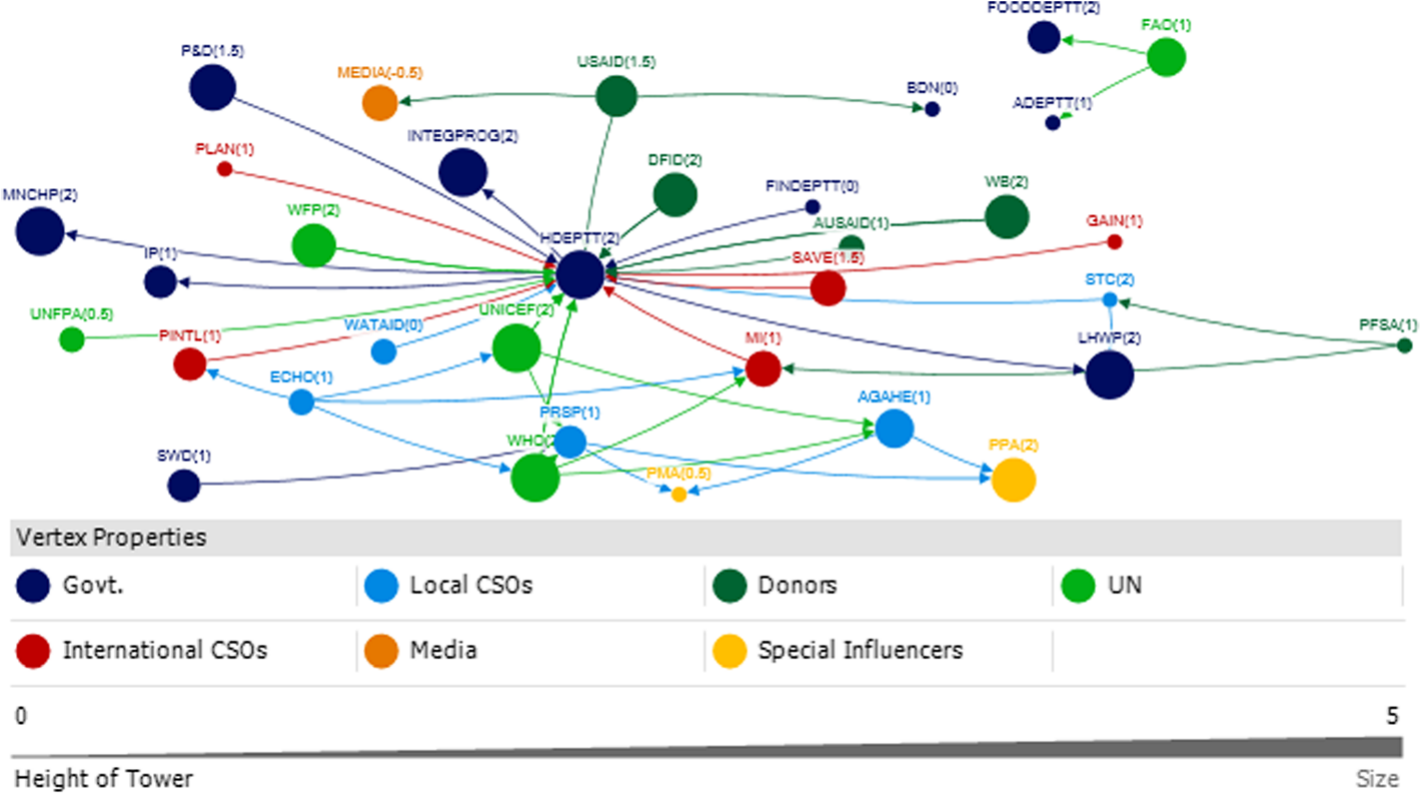
Punjab Funding Network

A total of 35 and 45 actors were identified with respect to funding links and technical links, respectively in the province of Punjab (Figs. 6 and 7).

### Punjab technical

The Health Department (HDEPTT) was the core actor which appeared central in the IYCF policy landscape in Punjab, with technical input provided by a variety of stakeholders including UN donors, INGOs, local NGOs, pediatricians and technical experts (Fig. 6). The Health Department received substantial technical support (in-degree), and was ranked most highly in terms of accessibility to a wide range of other actors in the network (closeness) (Table 4). HDEPTT also exerted a high degree of control over the flow of information between actors by its position in the network (betweenness). In discussions, participants indicated that the key focal point for IYCF policy at the provincial level was the Provincial Nutritional Strategy, which has IYCF as a core component, and also highlighted that they perceived there to be limited multi stakeholder involvement in policy making.

**Table 4:** Top 10 actors of technical and funding support networks in Punjab with respect to four network measures

PUNJAB								
Rank	Technical				Funding			
	In-degree	Out-degree	Between-ness	Closeness	In-degree	Out-degree	Between-ness	Closeness
1	13 HDEPTT	10 PED	754.457 PED	0.012 HDEPTT	17 HDEPTT	4 HDEPTT	758.767 HDEPTT	0.058 FAO
2	6 IP	7 PPA	712.119 HDEPTT	0.011 PED	3 MI	4 WHO	140.900 WHO	0.029 ADEPTT
3	4 ADEPTT	6 DFID	389.019 PPA	0.011 UNICEF	3 PRSP	4 ECHO	120.900 UNICEF	0.029 FOODDEPTT
4	4 TECHEXP	5 TECHEXP	360.905 IP	0.010 PPA	2 AGAHE	3 UNICEF	114.000 USAID	0.024 HDEPTT
5	4 UNICEF	4 PCPD	314.386 UNICEF	0.010 WHO	2 LHWP	3 USAID	111.500 PRSP	0.018 WHO
6	4 WHO	3 UNICEF	182.043 DFID	0.009 IP	2 PMA	2 PRSP	53.500 AGAHE	0.017 UNICEF
7	3 PHED	3 WHO	166.000 TRF	0.009 DFID	2 PPA	2 AGAHE	44.967 MI	0.016 MI
8	2 AGAHE	3 PHARMA	166.000 PFA	0.009 TECHEXP	1 ADEPTT	2 STC	21.333 STC	0.015 USAID
9	2 FOODDEPTT	3 TRF	133.967 ADEPTT	0.009 PHED	1 BDN	2 FAO	10.833 ECHO	0.015 STC
10	2 MEDIA	3 USAID	115.957 TECHEXP	0.009 MI	1 FOODDEPTT	2 PFSA	9.900 PINTL	0.014 PINTL

Refer to list of abbreviations

Participants indicated that UNICEF, technical experts, pediatricians and WHO were key influencers of IYCF policy and programs as providers of technical support mainly to the Health Department and the integrated program (a program formed by integration of the MNCH, LHW and Nutrition program) (Fig. 6). UNICEF and technical experts were the two actors who were ranked in the top ten for all network measures; these actors thus acted as well-connected (closeness and betweenness) hubs for information (both in-degree and out-degree) (Table 4).

It was also observed that even though the media and the breast milk substitute and baby food manufacturers/ pharmaceutical industry were influential in shaping the IYCF policy landscape; however, they were not supportive of the agenda (Fig. 6).

Participants of the net map activity also revealed that among the key academic actors which have played a role are Agriculture University Faisalabad, National Institute of Food Science and Technology, King Edward Medical University and University of Veterinary and Animal Sciences. These have been grouped under the technical experts who comprise the academia and research based entities in the net map Fig 6.

### Punjab funding

With funding obtained from 17 actors, the Health department was identified as the central actor in the funding network as well in the province (Fig. 7). HDEPTT was a significant receiver and dispenser of funding (in-degree and out-degree), and exerted a high degree of control over the flow of funds between actors by its position in the network (betweenness) (Table 4). In discussions, participants pointed out that although it was the health department that has the most influential network position, there were certain variations. In some areas, the development partners were funding the local NGOs to do the work and in other areas, it was the health department which was receiving the funds and dispersing them to the program. It was also noted that previously, policies had been developed unrealistically with limited budget allocations, and were not implemented. In such a restricted budget, IYCF was easily ignored as it had never been a priority. Additionally, provincial preferences have been leading to a different focus on nutrition.

The health department received funds mainly from UNICEF, WHO DFID and the World Bank (Fig. 7), with UNICEF and WHO most influential as providers of funds (out-degree) and mediators of the network (Table 4). It was highlighted by participants that the Integrated Program between 2013 and 2016 was receiving 70% of the total fund from the government and 30% from other donors (22 in all).

### Actor communities (punjab)

**Fig. 8:**
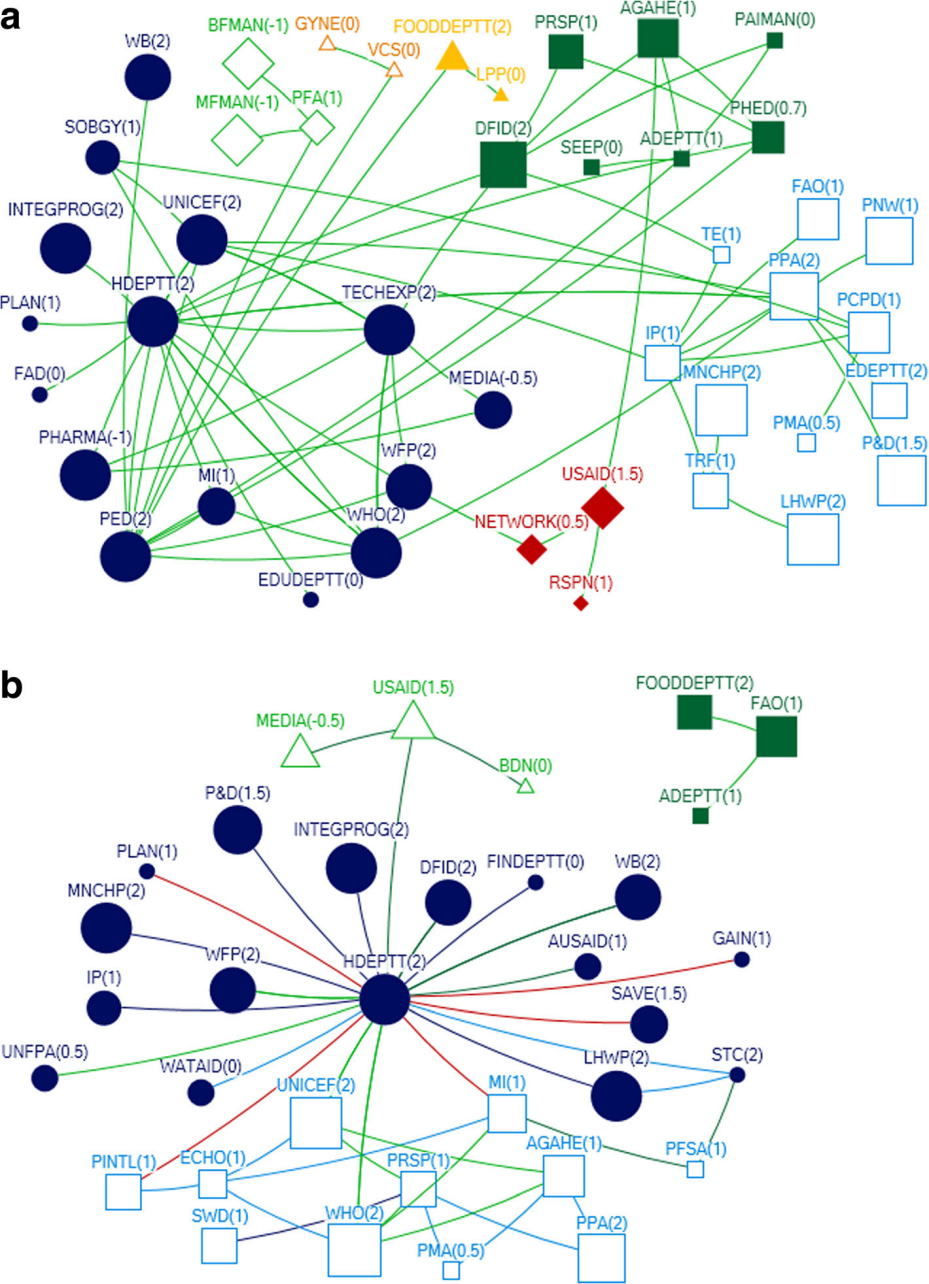
Punjab Technical and Funding Communities

There were three major communities identified in terms of the technical links in Punjab and two large communities identified in funding network (Fig. 8). Each of these communities comprised of a mix of different categories of actors. It is notable that Agricultural actors have a very separate funding community relevant to IYCF policy, separate to those for health. It can also be observed that the pharmaceutical industry and media were identified as part of the largest technical community in terms of the IYCF policy environment in Punjab.

### Sindh

The key actors identified in Sindh were from the government (i.e. Nutrition Cell Health Department) and development partners (i.e. WHO, UNICEF). (Table 5).

**Table 5:** Top 10 actors in technical and funding support networks with respect to four network measures in Sindh

Sindh								
Rank	Technical				Funding			
	In-degree	Out-degree	Between-ness	Closeness	In-degree	Out-degree	Between-ness	Closeness
1	11 UNICEF	11 UNICEF	748.000 UNICEF	0.014 UNICEF	3 HRSU	5 WHO	41.333 WHO	0.067 WHO
2	7 H DEPTT	2 HRSU	634.000 H DEPTT	0.014 PPA	3 N CELL	3 UNICEF	20.667 PN&D	0.059 PN&D
3	2 HRSU	7 H DEPTT	628.000 PPA	0.013 H DEPTT	3 PN&D	3 WB	20 AKU	0.056 N CELL
4	2 N CELL	1 PPA	354.000 HRSU	0.011 HRSU	2 AKU	1 AKU	10.333 UNICEF	0.056 H DEPTT
5	1 AKHSP	1 AKHSP	126.000 MANF	0.010 MANF	2 H DEPTT	1 H DEPTT	7 N CELL	0.053 UNICEF
6	1 e-MEDIA	1 WFP		0.010 MERLIN	1 PMDC	1 NPPI	6.333 H DEPTT	0.05 AKU
7	1 EPI			0.010 AKHSP	1 WHO	1 US AID	4.333 WB	0.05 WB
8	1 H EDU CELL			0.010 WFP			4 HRSU	0.05 HRSU
9	1 LHW PROG			0.010 ACF				0.042 NPPI
10	1 MNCH			0.045 USAID				0.042 PMDC

Refer to list of abbreviations

### Sindh technical

**Fig. 9:**
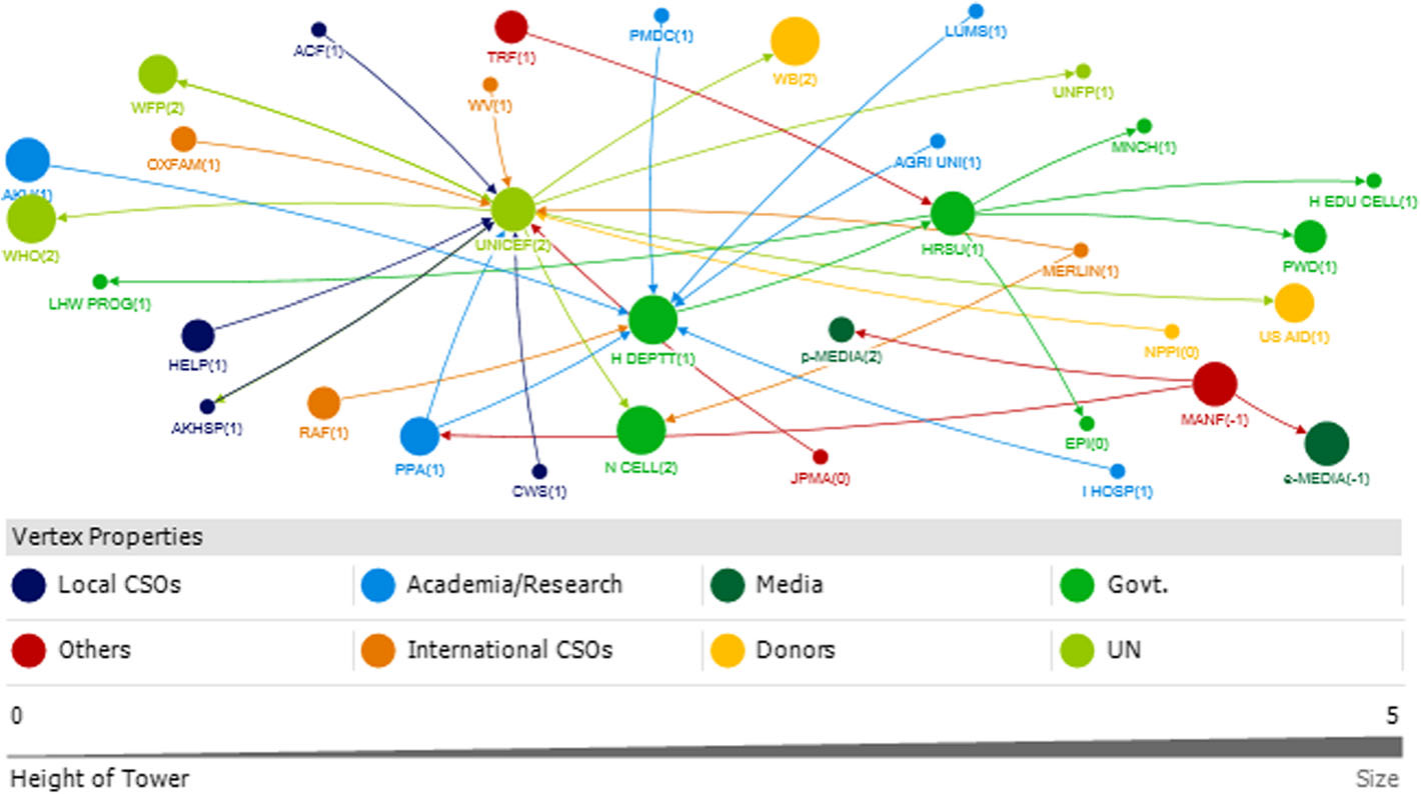
Sindh Technical Links

UNICEF, the Health Department, and the Health System Reform Unit were all very influential with respect to technical support (Fig. 9). These actors all received substantial technical support (in-degree), provided technical support (out-degree), were accessible to a wide range of other actors in the network (closeness), and exerted a high degree of control over the flow of information between actors by their positions in the network (betweenness) (Table 5).

The high level of influence by UNICEF with respect to technical support is partly derived from its role as a hub for technical IYCF support, for other donors and international agencies. One of the participants that the UN agencies (i.e. UNICEF, WHO and WFP) concluded that they will not work vertically, but together as a group after the Paris Declaration which is a practical, action oriented roadmap to improve the quality of aid and in turn improve impact on development [33].

Media was identified to play a dual role. On one hand it promotes breastfeeding in response to government advocacy. On the other hand, it helps to promote formula milk and baby food, which was seen as contradictory since formula milk and baby food manufacturers were identified as having conflicting agendas in the IYCF context Fig. 9.

### Sindh funding

WHO, the Health Department and Aga Khan University (AKU) were identified as the most influential actors with respect to funding in Sindh. They all ranked in the top ten as receivers of funding (in-degree), funders (out-degree), and as accessible (closeness) and exerting a high degree of control over the flow of funds (betweenness) (Table 5).

**Fig. 10:**
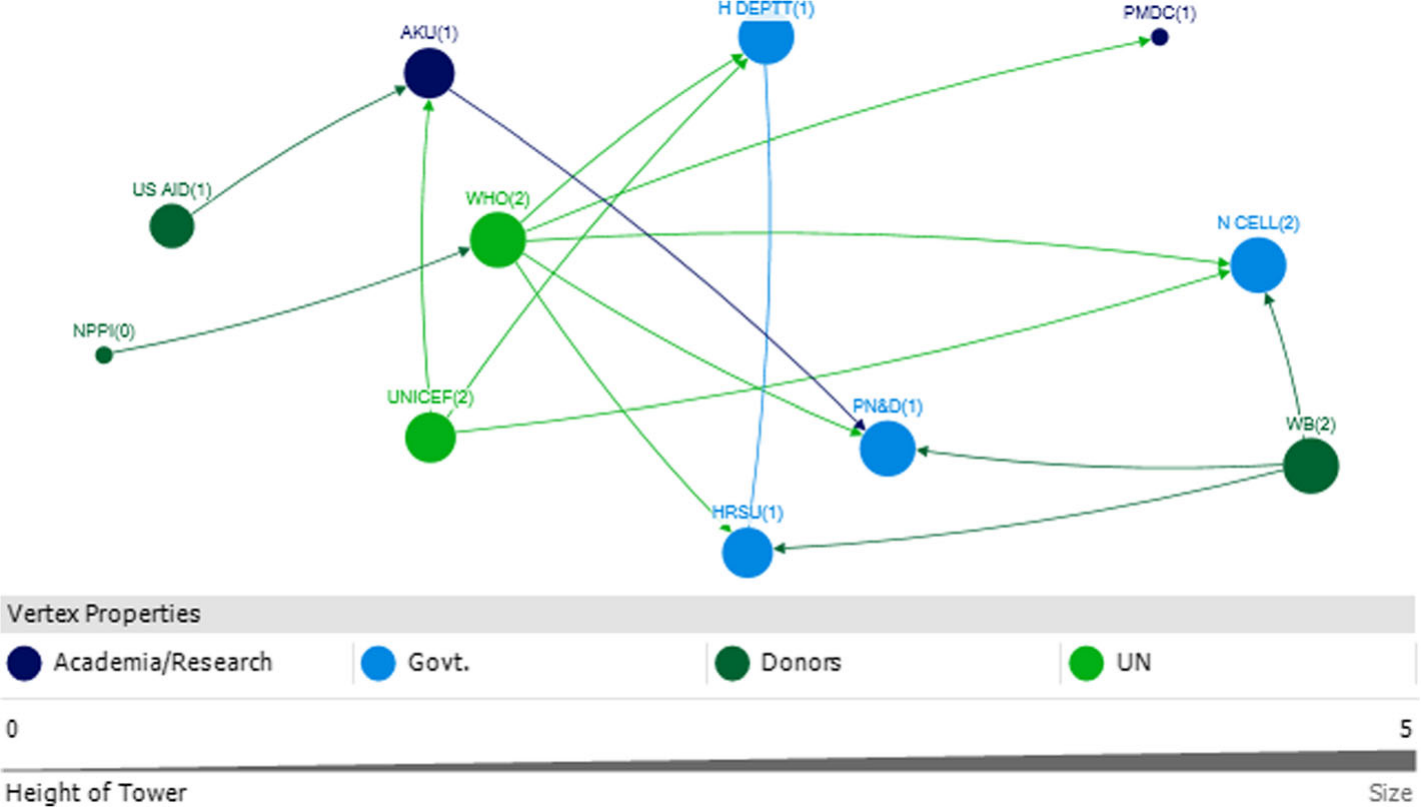
Sindh Funding Links

Other major donors were UNICEF and the World Bank; they were key donors to the Health Sector Reform Unit and the Nutrition Cell for the IYCF landscape in Sindh (Fig. 10).

### Actor communities (Sindh)

**Fig. 11:**
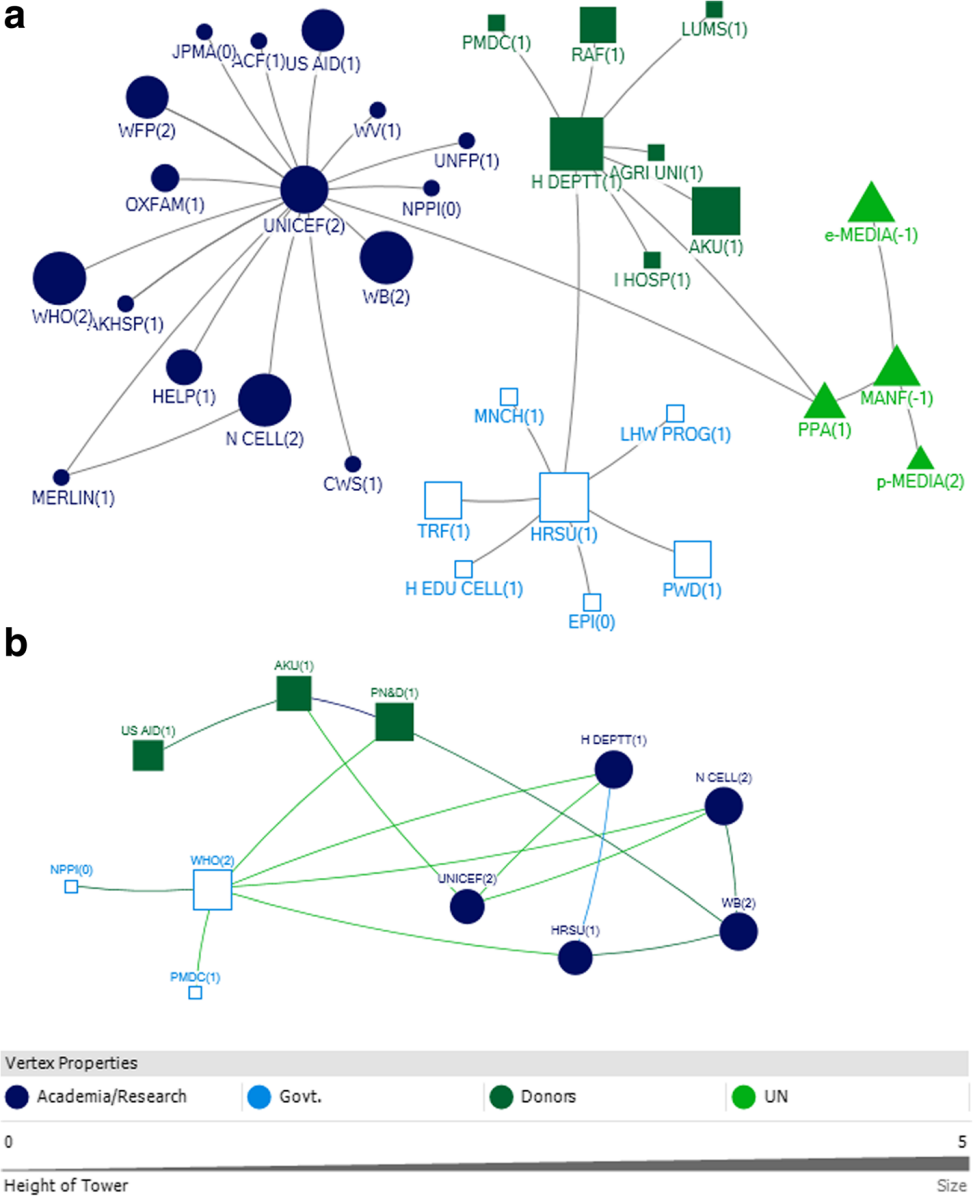
Sindh Technical and Funding Communities

There were four communities identified in the Sindh technical links (Fig. 11). The largest community, as indicated by the blue circles, was comprised of the majorly of development partners, INGOs and the Nutrition Cell as the only government partner. The Breast Milk Substitute and Baby Food Manufacturers/Pharmaceutical Industry developed another community with the Media and the Pakistan Pediatric Association. This was different when compared with Punjab, where the baby food manufacturers and media belonged to the largest community which included development partners and government.

There were also three communities identified in the funding links in Sindh (Fig. 11). The largest community (blue circles) comprised of the Health Department, Nutrition Cell, World Bank, Health Sector Reform Unit and UNICEF.

It was identified that the number of actors involved in the funding network were far less in Sindh as compared to Punjab. Whereas UNICEF and WHO were the major funders in Sindh contributing to the government actors, the health department in Punjab received funds from a number of other development partners and international NGOs.

## Conclusions

Overall, our analysis indicates strong policy support for IYCF in Pakistan. Many of these policies have been formulated under the influence of the global IYCF policy environment, and particularly with reference to WHO and UNICEF recommendations [34]. For example, the National IYCF strategy was developed under the Global IYCF strategy along with the Baby Friendly Hospital Initiative [35, 36]. Similarly, the Protection of Breastfeeding and Child Nutrition Ordinance reflects the International Code of Marketing of Breast milk Substitutes and the National Cluster Preparedness Response was formulated under the Operational Guidance on Infant Feeding in Emergencies [37–39].

The Pakistani IYCF policy environment is somewhat different from other South Asian countries where policy making and implementation is confined at a central level as opposed to provincial level in Pakistan. There may also be differences when considering the relatively high level of influence of UN actors, such as UNICEF and WHO on IYCF policy environment in Pakistan, compared to India and Sri Lanka [40, 41].

### Limitations of the research

Although the research was carefully prepared, there are certain limitations which can be addressed. One of the limitations in content analysis is that there is a possibility that not all documents have been included in this study either due to non-availability or the research team not being aware of them, although a thorough search took place. Future studies are recommended to cover any missed documents along with new documents generated after 2014. Similarly, although for each Net-Map activity we invited uniform representation from all the categories, but there was limited participation from the government sector, either health or non-health.

### Challenges and opportunities

The policy content analysis reported here shows that there have been certain policies, which have been developed at the national level, that have yet to be translated into the provincial level to provide impactful implementation. These include Pakistan Integrated Nutrition Strategy, National Infant and Young Child Feeding Strategy, and the National Nutrition Cluster Preparedness response. Similarly, stakeholder analysis identified that the number of actors involved in the funding network were far less in Sindh as compared to Punjab. Whereas UNICEF and WHO were the major funders in Sindh contributing to the government actors, the Health Department in Punjab received funds from a number of other development partners and international NGOs. In terms of the technical links, it was the Health Department in both the provinces who received technical input from a variety of stakeholders which in case of the federal level was the National Nutrition Wing.

Although due to devolution of the Health ministry in Pakistan there has been change in the scenario, analysis of the IYCF policy environment indicates some positive trends. Very recently (2016), the Pakistan National IYCF strategy has been endorsed by the Ministry of National Health Services Regulations and Coordination with National IYCF guidelines development and communication strategy underway. Additionally, the development of certain provincial level policies including the breastfeeding acts, Nutrition policy guidance notes, PC-1 s and maternity leave acts indicates that these provinces have started to take up the responsibility of strengthening their systems, although there is more to be done in those documents as indicated by the following key concerns.

### Lack of ownership

The content analysis and stakeholder analysis show that policies supporting IYCF in Pakistan are developed by various bodies including the ministries of health, Planning and Development, and agriculture at the federal and/or provincial level. Although the rest of the ministries continue to have federal level health policy making, it is the devolution of only the health ministry which has led to transitional problems in health policy development and implementation. Before devolution of the Ministry of Health, policies were developed at national level and were used as reference for implementation across all the provinces, with a national body monitoring the process. However, after devolution, the provinces have become autonomous with respect to health policy, with each adapting and modifying the nationally made policies or strategies per their context being responsible for their own implementation. This change in practice by involvement of relevant stakeholders during decision making process will improve the sense of ownership by the provinces which are the real implementers.

### Inadequate multisectoral collaboration

IYCF policy development encompasses a wide range of sectors such as agriculture and food security, legislative bodies, consumer protection, education, and women’s affairs [25, 30]. With different institutional bodies involved in making these policies in silos, there has been a gap observed in multisectoral collaboration. Our stakeholder analysis indicated that actors within health perceived inadequate involvement of other relevant stakeholders. Each body has highlighted the importance of IYCF, either directly or indirectly, but inter sectoral collaboration has not been highlighted as a key strategy.

It is therefore recommended to expand the multi-sectoral national and provincial IYCF committees so that they include representatives from government departments, NGOs, development partners, healthcare professionals, education, agriculture and academia. The multi-sectoral plans endorsed by the governments of Nepal and Sri Lanka can be modified for the Pakistani context. Additionally, it can also be beneficial to make practical knowledge on IYCF, across the multi-sectoral landscape, more accessible and coherent. Formulating a proper body for undertaking the monitoring and evaluation of the implementation of these policies would also be advantageous. All of these recommendations can provide detailed information in policies on achieving objectives and avoiding duplication of information.

### Inadequate training guidelines

As indicated earlier, the training and capacity building of community health workers to deliver IYCF support and intervention, is an integral part of successful policy implementation. This content analysis and Net-Mapping highlighted an opportunity to support implementation of existing policies for training of community health workers. This support has been highlighted through specific training guidelines that are referenced in these policies; however, these guidelines are inadequate and require more in depth guidance on proper implementation. Except for the guidelines indicated in the National Program for Family Planning and Primary Health Care, the rest of the policies provide inadequate training guidelines. It is recommended to develop training guidelines by consensus with all stakeholders and to uniformly implement them throughout the country with defined minimum standards of training identified.

### Lack of clearly defined roles and responsibilities

Participants of the stakeholder analysis indicated that although financial commitments have been observed by donors to assist in implementation of policies, their roles and responsibilities have not been clearly defined. This can be improved by developing national and provincial plans of action and the mobilization of related resources, coupled with advocacy to provide information to key stakeholders. This requires choosing context appropriate actions and also implementing them at scale. Additionally, developing an information base on ongoing IYCF related issues and strategies would enhance the effectiveness of policy and programme decisions by policy-makers, managers, and related staff, and would also require developing national and provincial coordinators for infant and young child feeding so as to oversee the sustainability of programs related to these policies.

## Abbreviations

ACF: Action against hunger
ADEPTT: Agriculture Department
AGRI UNI: Agriculture University
AKHSP: Agha Khan Health Services Pakistan
AKU: Agha Khan University
AUSAID: Australian Agency for International Development
BDN: Basic Development needs
DFID: Department for International Development
ECHO: Echo Med
e-Media: Electronic media
EPI: Extended programme for immunization
FAO: Food and Agriculture Organization
FOODDEPTT: Food Department
H EDU CELL: Health education cell
HDEPT: Health Department
HRSU: Health system reforms unit
IFPRI: International Food Policy Research Institute
IP: Integrated program
IYCF: Infant and young child feeding
LAWMIN: Ministry of Law
LHWP: Lady health workers program
MANF: Manufacturers
MEDIA: MEDIA
MERLIN: Merlin
MI: Micronutrient initiative
MNCH: Maternal Neonatal Child Health
MNCHP: Maternal Neonatal Child Health Program
MOH: Ministry of Health
MOPW: Ministry of Population Welfare
NCELL: Nutrition cell
NETWRK: The network
NNW: National nutrition wing
NPPI: Norway Pakistan partnership initiative
ORA: Organizational risk analyzer
P&D: Planning & S
PARL: Parliamentarians
PCPD: Punjab Consumer Protection Department
PED: Pediatric professors
PFSA: Punjab Forensic Science Agency in Punjab
PHARMA: Pharmaceuticals
PHED: Public Health Engineering Department
PINTL: Plan International
PMA: Pakistan Medical Association
PMDC: Pakistan Medical & Dental Council
PN&D: Planning & Development Department
PPA: Pakistan Pediatric Association
PRSP: Punjab rural support program
SAIFRN: South Asian Infant Feeding Research Network
SAVE/STC: Save the children
TECHEXP: Technical experts
TRF: Technical resource facility
UNICEF: United Nation’s International Emergency Fund
USAID: United States Agency for International Development
WB: World Bank
WFP: World Food Program
WHO: World Health Organization
